# Supplementary material for: Gas adsorption and framework flexibility of CALF-20 explored via experiments and simulations
Source: Nat Commun. 2024 May 9;15:3898. doi: 10.1038/s41467-024-48136-0 (PMC11081952; doi:10.1038/s41467-024-48136-0)
Supplement: Supplementary file 1 — Supplementary Information [file 41467_2024_48136_MOESM1_ESM.pdf]

Supplementary Information

Gas adsorption and framework flexibility of CALF-20 explored via experiments and simulations

Rama Oktavian[1‡], Ruben Goeminne[2‡], Lawson T. Glasby[1], Ping Song[3], Racheal Huynh[3], Omid Taheri Qazvini[3], Omid Ghaffari-Nik[3], Nima Masoumifard[3], Joan L. Cordiner[1], Pierre Hovington[3], Veronique Van Speybroeck[2], Peyman Z. Moghadam[4*]

[1]*Department of Chemical and Biological Engineering, The University of Sheffield, Sheffield S1 3JD, UK*

[2]*Center for Molecular Modeling (CMM), Ghent University, Technologiepark 46, 9052 Zwijnaarde, Belgium*

[3]*Svante Inc., 8800 Glenlyon Pkwy, Burnaby, BC Canada V5J 5K3*

[4]*Department of Chemical Engineering, University College London, London WC1E 7JE, UK*

*Email: p.moghadam@ucl.ac.uk

Supplementary Methods 1

Powder X-ray Diffraction (PXRD) measurements

PXRD patterns were collected using a Rigaku Miniflex 600 bench top PXRD equipped with a CuK_α x-ray source. The samples were prepared by soaking the powders in a solvent (ethanol or water) for 1 hour and then drop casted onto a standard sample holder. The PXRD pattern was collected while the powder was still wet. For the samples run under N_2, the powder was loaded onto a BTS 500 high temperature sample holder and heated to 110°C for 45 minutes under constant N_2 flow. Then the powder was allowed to cool to 50°C and equilibrated at this temperature for 10 minutes. The PXRD pattern was then collected at 50°C. The samples run under CO_2 were dried under N_2 and then during the PXRD collection, 100% CO_2 was flowed through under the same conditions described above. The PXRD patterns under different relative humidities (%RH) were collected by flowing wet gas through the BTS 500 chamber. The humidity of wet gas was controlled using gas bubbler and monitored with a RH probe.

Gas adsorption measurements

Nitrogen and CO_2 isotherms were measured using ASAP2020 from Micromeritics Instruments Inc. Before gas adsorption, the samples were heated under vacuum at 140 °C for 5 hours. The water isotherm was measured using a gravimetric vapor sorption analyzer (DVS Resolution, Surface measurement system). Samples were activated under dry nitrogen flow at 140 °C for 2 hours. Dm/dt value of 0.0005%/min was used as the equilibrium criteria for each data point.

Supplementary Figures

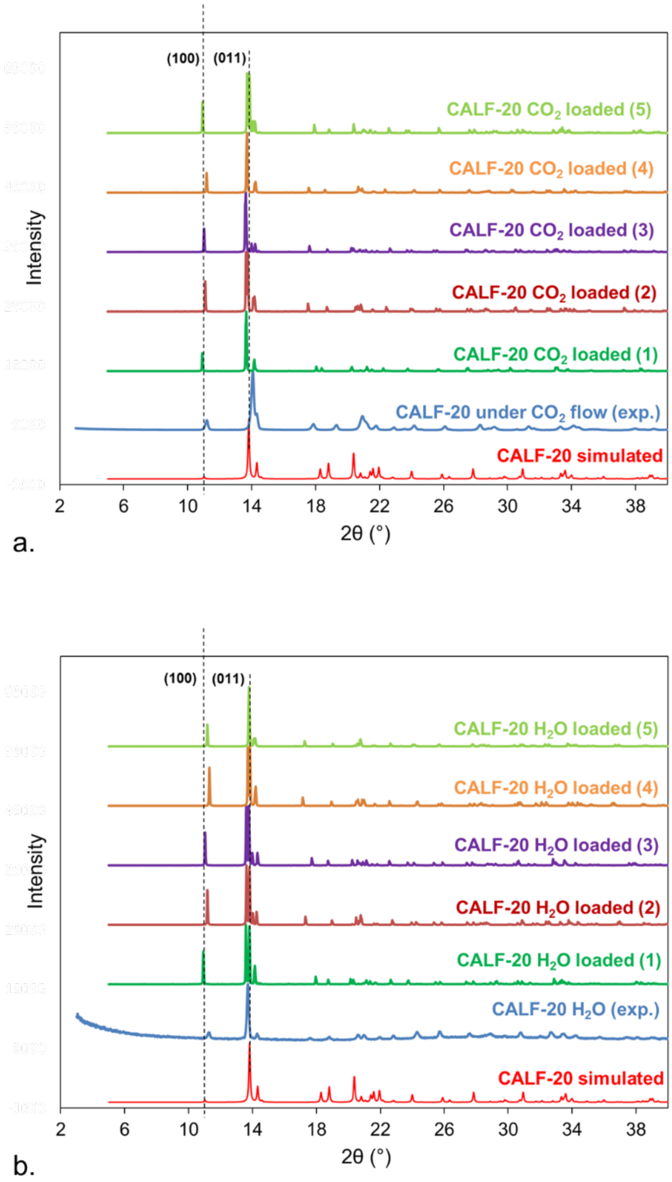

Supplementary Fig. 1. The powder X-ray diffraction (PXRD) patterns for **a.** CO_2-induced CALF-20 and **b.** water-induced CALF-20 obtained from different configuration of NPT simulations. The simulated PXRDs are compared with experimental PXRDs in the presence CO_2 and soaked in water.

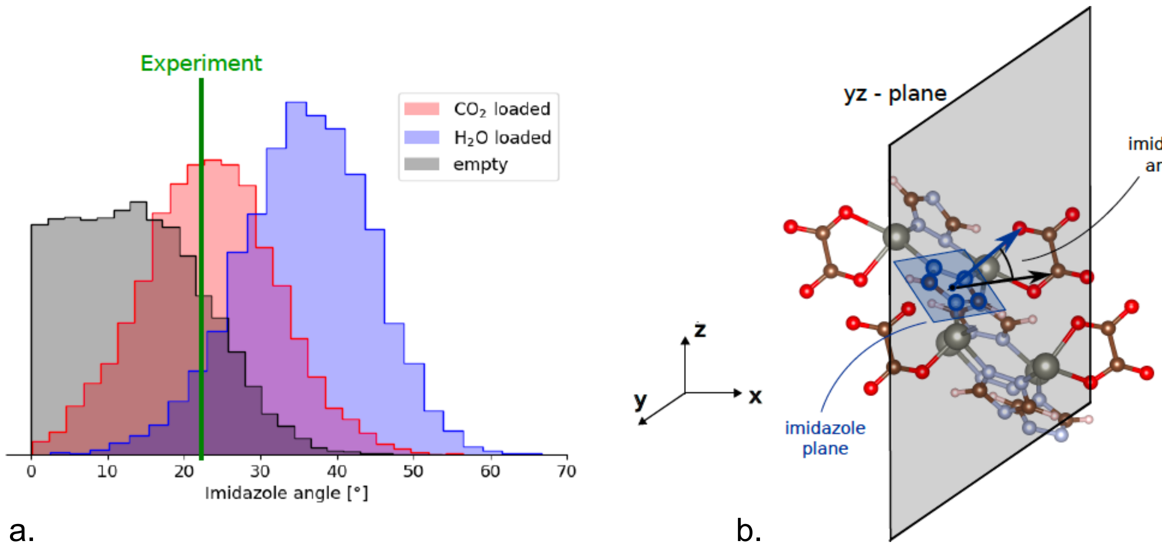

Supplementary Fig. 2. a. Histograms showing the rotation of the traizolate linkers in the experimental, empty, CO_2-loaded and H_2O-loaded CALF-20 structures. The angle distributions are obtained from the free energy profiles at saturation loadings and 273 K. **b.** Schematic showing the angle between the normal on the plane defined by the triazolate linker and the YZ-plane calculated in **a.**

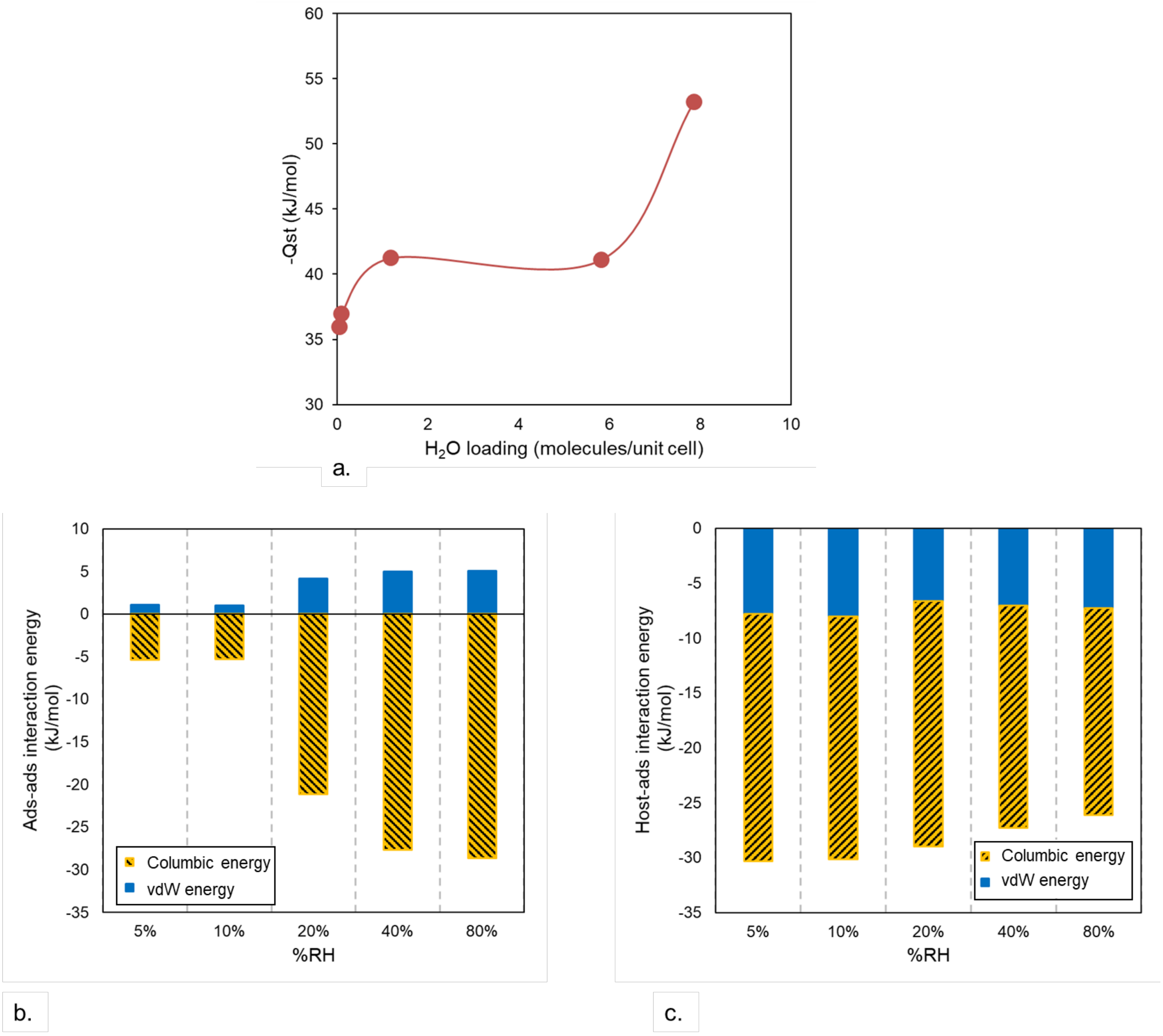

Supplementary Fig. 3. a. Heat of adsorption versus water loading in molecules per unit cells of CALF-20 at 293 K. Breakdown of van der Waals and Columbic interaction energy for **b.** water-water and **c.** water-framework at different relative humidities (%RH).

Supplementary Methods 2

Gas adsorption isotherms in CALF-20 were calculated via grand canonical Monte Carlo (GCMC) simulations using the RASPA simulation software.[1] The Lennard-Jones (LJ) parameters for adsorbent atoms are tabulated in Table S1. N_2 and CO_2 molecules were modelled using the TraPPE[2] force field with charges placed on each atom and at the centre of mass. Water molecule was modelled using the TIP4P force field.[3] Table S2-S4 tabulates all of the LJ parameters and atomic charges for all gas molecules.

Supplementary Tables

Supplementary Table 1. LJ parameters for CALF-20

**Atom type**	**σ (Å)**	**ε/k$_B$ (K)**	**Force field**
**C**	3.473	47.859	Dreiding[4]
**O**	3.033	48.160	Dreiding[4]
**H**	2.846	7.649	Dreiding[4]
**N**	3.263	38.951	Dreiding[4]
**Zn**	4.045	27.677	Dreiding[4]

Supplementary Table 2. LJ parameters and charges for N_2

**Atom type**	**σ (Å)**	**ε/k$_B$ (K)**	**Atomic charge**	**Force field**
**N_N$_2$**	3.31	36.0	-0.482	TraPPE[2]
**N_center of mass**	0	0	0.964	TraPPE[2]
**N_N$_2$**	3.31	36.0	-0.482	TraPPE[2]

Supplementary Table 3. LJ parameters and charges for CO_2

**Atom type**	**σ (Å)**	**ε/k$_B$ (K)**	**Atomic charge**	**Force field**
**C_CO$_2$**	2.80	27.0	0.70	TraPPE[2]
**O_CO$_2$**	3.05	79.0	-0.35	TraPPE[2]

Supplementary Table 4. LJ parameters and charges for water

**Atom type**	**σ (Å)**	**ε/k$_B$ (K)**	**Atomic charge**	**Force field**
**O_H$_2$O**	3.154	78.0	0	Tip4P[3]
**H_H$_2$O**	0	0	0.52	Tip4P[3]
**M_H$_2$O**	0	0	-1.04	Tip4P[3]

Supplementary References

(1) Dubbeldam, D.; Calero, S.; Ellis, D. E.; Snurr, R. Q. RASPA: Molecular Simulation Software for Adsorption and Diffusion in Flexible Nanoporous Materials. *Mol. Simul.* **2016**, *42* (2), 81–101. https://doi.org/10.1080/08927022.2015.1010082.

(2) Potoff, J. J.; Siepmann, J. I. Vapor–Liquid Equilibria of Mixtures Containing Alkanes, Carbon Dioxide, and Nitrogen. *AIChE J.* **2001**, *47* (7), 1676–1682. https://doi.org/10.1002/aic.690470719.

(3) Vega, C.; Abascal, J. L. F.; Nezbeda, I. Vapor-Liquid Equilibria from the Triple Point up to the Critical Point for the New Generation of TIP4P-like Models: TIP4P/Ew, TIP4P/2005, and TIP4P/Ice. *J. Chem. Phys.* **2006**, *125* (3), 34503. https://doi.org/10.1063/1.2215612.

(4) Mayo, S. L.; Olafson, B. D.; Goddard, W. A. DREIDING: A Generic Force Field for Molecular Simulations. *J. Phys. Chem.* **1990**, *94* (26), 8897–8909. https://doi.org/10.1021/j100389a010.
